# Alanine aminotransferase elevation in hospitalized children with infectious mononucleosis: independent associations with Epstein–Barr virus DNA load, age, and sex

**DOI:** 10.3389/fped.2026.1901064

**Published:** 2026-07-07

**Authors:** Songlin Gan, Yuan Tang, Lianying Jiang, Huazhuo Mai

**Affiliations:** Department of Pediatrics, Maoming People's Hospital, Maoming, China

**Keywords:** age groups, alanine aminotransferase elevation, Epstein–Barr virus, infectious mononucleosis, viral load

## Abstract

**Background:**

Alanine aminotransferase (ALT) elevation is common in pediatric infectious mononucleosis, but its relationship with Epstein–Barr virus (EBV) DNA load, age, and sex remains incompletely defined. We examined this association and identified independent correlates across pediatric age groups.

**Methods:**

In this single-center retrospective cross-sectional study, we included 365 children hospitalized with EBV-associated infectious mononucleosis who had admission EBV DNA quantification. Patients were grouped by age as <3, 3–6, and ≥6 years. Multivariable linear regression was used to evaluate the association between EBV DNA load and ALT level, while multivariable logistic regression assessed factors associated with ALT elevation. Age-stratified and interaction analyses examined whether these associations differed by age group, and sensitivity analyses tested the stability of the findings.

**Results:**

Higher log10(EBV DNA) was associated with higher ln(ALT) in the fully adjusted model [adjusted *β* = 0.32, 95% confidence interval (CI): 0.24–0.41; *P* < 0.001], corresponding to an approximately 38% higher ALT level for each 10-fold increase in EBV DNA load. In the primary logistic model, EBV DNA load, age, and sex were associated with ALT elevation. Compared with children aged <3 years, the adjusted odds ratios were 2.08 (95% CI: 1.15–3.74) for those aged 3–6 years and 5.89 (95% CI: 3.04–11.42) for those aged ≥6 years. Male sex was associated with lower odds of ALT elevation (adjusted odds ratio: 0.29, 95% CI: 0.17–0.51). The EBV DNA load–ALT association was similar across age groups and remained stable in sensitivity analyses.

**Conclusions:**

Among hospitalized children with EBV-associated IM, higher EBV DNA load, older age, and female sex were independently associated with ALT elevation, findings that may help contextualize ALT elevation at initial evaluation.

## Introduction

Epstein–Barr virus (EBV) infects over 90% of the global population, with primary infection occurring predominantly in childhood and adolescence (1–4). Among immunocompetent children, primary EBV infection may manifest as infectious mononucleosis (IM), characterized by fever, pharyngotonsillitis, cervical lymphadenopathy, and atypical lymphocytosis (5–7). IM manifestations vary by age, younger children often present with nonspecific symptoms, while school-age children and adolescents show more typical symptoms and laboratory abnormalities (5, 8, 9).

Hepatic involvement is common in pediatric IM, most often detected as alanine aminotransferase (ALT) elevation, although ALT reflects hepatocellular injury rather than clinically defined hepatitis (10, 11). EBV-related hepatic involvement is usually mild and self-limited, but a minority of patients may develop hepatitis, cholestatic features, or, rarely, fulminant hepatic failure (12–14). Hepatic injury in acute IM is thought to be predominantly immune-mediated (1), although higher EBV DNA load has been associated with greater disease burden, including more marked hepatic dysfunction, broader immune perturbation, and longer hospitalization (15, 16).

Real-time polymerase chain reaction (PCR) quantification of EBV DNA provides a useful measure of viral load in pediatric IM, especially when serology is inconclusive (17–19). Older children more often present with typical IM symptoms and greater biochemical evidence of hepatic involvement, while younger children tend to present less typically and with milder biochemical abnormalities (5, 8, 9). Sex differences have been reported in the immune responses and clinical manifestations of other viral infections (20, 21), but evidence on sex differences in ALT elevation or hepatic involvement in pediatric EBV-associated IM remains uncertain (10, 22, 23).

However, the quantitative association between EBV DNA load and ALT, and whether this relationship differs by age group, remains incompletely characterized. Recent pediatric IM cohorts have reported associations between higher EBV DNA load and elevated liver enzymes or hepatic injury markers (15, 16, 22), but none formally evaluated whether the EBV DNA load–ALT relationship differs by age group.

To address these gaps, we performed a single-center, retrospective cross-sectional analysis of 365 hospitalized children with EBV-associated IM and EBV DNA load measured at admission. Using multivariable linear regression with sequential covariate adjustment, we estimated the association between EBV DNA load and ALT and identified covariates, including age group and sex, independently associated with ALT elevation. We further assessed whether this association differed by age group and conducted prespecified sensitivity analyses to evaluate robustness.

## Materials and methods

### Study design and population

**Figure 1:**
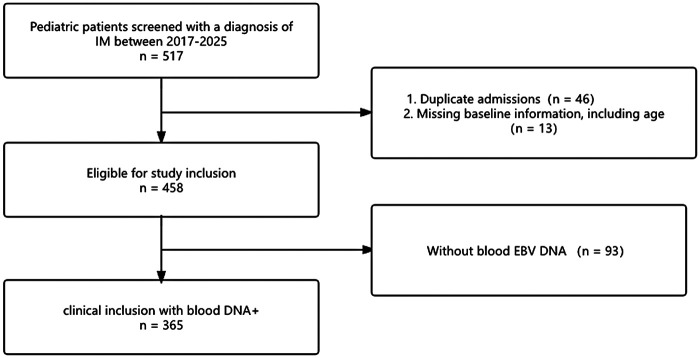
Study participant flow diagram. Children hospitalized with EBV-associated infectious mononucleosis between 2017 and 2025 were screened, and 365 were included in the final analytical cohort.

This single-center, retrospective, cross-sectional study examined the association between admission EBV DNA load and concurrent ALT level. We reviewed the medical records of children hospitalized with infectious mononucleosis (IM) at our institution between 2017 and 2025. Children were eligible if they had EBV-associated IM and quantitative blood EBV DNA and ALT measurements at admission. For children with more than one eligible hospitalization, only the first was included. The final cohort comprised 365 children (Figure 1). All primary analysis variables were complete, and no further exclusions for missing data were applied.

### Diagnostic criteria

EBV-associated IM was diagnosed according to the expert consensus on the diagnosis and treatment of EBV infection-related diseases in children (24). The diagnosis required at least three of the following clinical manifestations: fever, pharyngotonsillitis, cervical lymphadenopathy, hepatomegaly, splenomegaly, and eyelid edema. Laboratory evidence included evidence of primary EBV infection, defined as positive anti-EBV viral capsid antigen (VCA)-IgM and anti-EBV-VCA-IgG with negative anti-EBV nuclear antigen (EBNA)-IgG, or isolated anti-EBV-VCA-IgG positivity with low VCA-IgG avidity. In accordance with the consensus, supportive non-specific laboratory evidence included a peripheral-blood atypical lymphocyte proportion of ≥10% on blood smear. To avoid duplicate counting, only the first eligible hospitalization per child was retained. The study cohort was further restricted to children with quantitative peripheral-blood EBV DNA results available at admission.

Age groups were prespecified before analysis. Age was recorded at admission in years to one decimal place and grouped into three non-overlapping categories: <3, 3–6, and ≥6 years. For analytical coding, the 3–6 stratum included children aged 3.0–<6.0 years. These categories were intended to approximate infancy/toddlerhood, preschool age, and school age, respectively, and were aligned with prior pediatric IM studies that stratified children by age when comparing clinical and laboratory features. The grouping was also clinically plausible because the presentation of primary EBV infection varies by age: younger children more often have mild or atypical disease, whereas school-age children more often present with a classic IM phenotype (5, 8, 25). Age was therefore evaluated as a prespecified effect modifier in the statistical models.

### Variable definitions

Serum ALT and EBV DNA load were measured at admission and analyzed as concurrent measurements from the index hospitalization. For the primary binary outcome, ALT elevation was defined as ALT >40 U/L, the institutional upper limit of normal (ULN) used for all pediatric age groups. ALT was used as a biochemical marker of hepatocellular injury, and the outcome is therefore described as ALT elevation rather than clinically defined hepatitis. Because pediatric ALT reference ranges vary by age and sex, sensitivity analyses redefined ALT elevation using age- and sex-specific pediatric ULNs. EBV DNA load was quantified in peripheral blood by real-time PCR and reported in copies/mL. Laboratory testing was performed as part of routine clinical care in the institutional clinical laboratory. Because of the retrospective, record-based design, platform-level assay details could not be reliably reconstructed across the full study period and therefore could not be reported uniformly. For regression analyses, ALT was natural log-transformed [ln(ALT)] and EBV DNA load was log10-transformed [log10(EBV DNA)] to reduce the influence of right-skewed distributions. Age group and sex were prespecified covariates in the primary adjusted models; pre-hospital fever days and pre-admission corticosteroid use (yes/no) were included as additional covariates in supplementary models. No imputation was performed.

### Statistical analysis

Continuous variables were assessed for normality using the Shapiro–Wilk test and histogram inspection. Normally distributed variables were presented as mean ± standard deviation (SD), skewed variables as median [interquartile range (IQR)], and categorical variables as *n* (%). Between-group comparisons used one-way ANOVA for normally distributed continuous variables, the Kruskal–Wallis test for skewed continuous variables, and the chi-square or Fisher exact test for categorical variables.

The association between log10(EBV DNA) and ln(ALT) was examined in three sequential linear regression models. Model 1 was unadjusted. Model 2 was adjusted for sex and age group, with female sex and age <3 years as reference categories. Model 3 was additionally adjusted for pre-hospital fever days. Regression coefficients are reported as *β*, representing the change in ln(ALT) per 1-unit increase in log10(EBV DNA).

Pre-hospital fever days were included only in the extended model because they may reflect disease progression rather than baseline confounding. Pre-admission corticosteroid use was excluded from the primary models because treatment decisions were likely driven by clinical severity, raising concern for confounding by indication; this variable was instead evaluated in sensitivity analyses.

Factors associated with ALT elevation (>40 U/L) were assessed in two logistic regression models. Model A, the parsimonious model, was adjusted for log10(EBV DNA), sex, and age group. Model B additionally included pre-hospital fever days and served as the primary logistic model. Female sex and age <3 years served as reference categories. Results are reported as odds ratios (ORs) with 95% confidence intervals (CIs). As a *post hoc* supportive analysis, ALT was also categorized by multiples of the ULN (≤1×, 1–5×, and >5×) and modeled as an ordered outcome using proportional-odds ordinal logistic regression with the same covariate structure as the primary models. As clinical-course outcomes, length of hospital stay and the number of in-hospital fever days (both counts) were modeled by negative binomial regression with EBV DNA load grouped using conventional thresholds (<10^4^, 10^4^–10^6^, and >10^6^ copies/mL; reference: <10^4^), adjusted for age group and sex, with rate ratios (RRs) and 95% CIs reported; differences across groups were also assessed by the Kruskal–Wallis test. To address potential temporal variation in PCR assays across the study period, a further sensitivity analysis additionally adjusted the primary linear model for admission period (2017–2021 vs. 2022–2025), repeated it within each period, and tested a period × log10(EBV DNA) interaction.

Age-stratified subgroup analyses were prespecified, and the age group × log10(EBV DNA) interaction was tested in both unadjusted and adjusted models using the likelihood ratio test. Four sets of sensitivity analyses were planned. First, the primary linear and logistic models were further adjusted for pre-admission corticosteroid use. Second, ALT elevation was redefined in logistic models using age- and sex-specific pediatric ULNs; the original covariate structure [log10(EBV DNA), sex, age group, and pre-hospital fever days] was retained for comparability, and a parsimonious model with log10(EBV DNA) and pre-hospital fever days only was additionally fitted because age and sex were already embedded in the alternative outcome definition. Third, the functional form of the age–ln(ALT) association was examined with restricted cubic splines (three knots) in a multivariable linear model adjusted for log10(EBV DNA), sex, and pre-hospital fever days; the overall association and departure from linearity were tested with the Wald test. Fourth, categorical age groups were replaced by continuous age in two parallel models: Model A adjusted for sex and age, and Model B additionally adjusted for pre-hospital fever days. These subgroup, spline, and alternative-outcome analyses were prespecified to assess robustness and are reported as supportive rather than confirmatory.

All tests were two-sided, and *P* < 0.05 was considered statistically significant. Because this was a retrospective study, no *a priori* sample-size calculation was performed. Analyses were performed in R version 4.2.2 (R Foundation for Statistical Computing, Vienna, Austria; http://www.R-project.org) and the Free Statistics analysis platform (version 2.2, Beijing, China).

## Results

### Baseline characteristics

A total of 365 children with EBV-associated IM were included (Table 1). The cohort was predominantly male (252/365, 69.0%), with no significant difference in sex distribution across age groups (*P* = 0.256). Serum ALT increased with age, with median values of 29.8 U/L (IQR: 19.1–73.4) in children aged <3 years, 64.6 U/L (IQR: 24.1–158.6) in those aged 3–6 years, and 85.8 U/L (IQR: 39.9–236.9) in those aged ≥6 years (*P* < 0.001). EBV DNA load also differed across age groups and was highest in children aged 3–6 years, with a median of 57,230 copies/mL (IQR: 5,106–256,650), followed by those aged ≥6 years, with a median of 29,185 copies/mL (IQR: 4,475–219,875), and those aged <3 years, with a median of 16,420 copies/mL (IQR: 500–159,900) (*P* = 0.008). Globulin levels increased with age (*P* < 0.001), whereas albumin (*P* = 0.045) and total protein (*P* < 0.001) also differed across groups. Pre-hospital fever days, white blood cell count, lymphocyte count, monocyte count, atypical lymphocyte percentage, and C-reactive protein (CRP) were similar across age groups (all *P* > 0.05).

**Table 1:** Baseline clinical and laboratory characteristics by age group.

Variable	Total (*n* = 365)	<3 years (*n* = 97)	3–6 years (*n* = 152)	≥6 years (*n* = 116)	*P* value
Sex, *n* (%)					0.256
Female	113 (31.0)	36 (37.1)	46 (30.3)	31 (26.7)	
Male	252 (69.0)	61 (62.9)	106 (69.7)	85 (73.3)	
Age (years)	—	1.8 (1.3–2.5)	4.8 (3.9–5.7)	8.5 (7.3–10.4)	<0.001
ALT (U/L)	56.7 (23.5–155.1)	29.8 (19.1–73.4)	64.6 (24.1–158.6)	85.8 (39.9–236.9)	<0.001
ALT elevation, *n* (%)	212 (58.1)	38 (39.2)	87 (57.2)	87 (75)	<0.001
EBV DNA (copies/mL)	29,770 (2,933–233,000)	16,420 (500–159,900)	57,230 (5,106–256,650)	29,185 (4,475–219,875)	0.008
WBC (×10^9^/L)	14.7 (11.5–19.6)	15.9 (11.0–20.5)	15.2 (12.5–19.5)	13.4 (11.4–18.6)	0.267
Lymphocyte count (×10^9^/L)	8.4 (6.4–12.3)	9.2 (5.7–14.0)	8.4 (6.9–12.1)	7.9 (5.8–11.5)	0.232
Monocyte count (×10^9^/L)	1.1 (0.8–2.0)	1.4 (0.8–2.2)	1.1 (0.8–1.9)	1.0 (0.7–1.7)	0.203
Atypical lymphocyte (%)	18.0 (11.0–30.0)	17.0 (12.0–25.0)	20.0 (11.0–32.0)	17.0 (11.0–30.0)	0.637
Total protein (g/L)	69.1 ± 5.5	67.7 ± 4.8	68.4 ± 5.1	71.1 ± 5.9	<0.001
Globulin (g/L)	30.3 (27.7–33.5)	28.6 (25.8–31.9)	29.9 (27.8–32.9)	32.3 (29.8–35.4)	<0.001
Albumin (g/L)	38.4 (36.1–40.7)	39.1 (37.2–41.0)	38.2 (35.8–40.7)	38.1 (35.7–40.3)	0.045
CRP (mg/L)	7.0 (5.0–13.9)	6.4 (5.0–11.8)	7.7 (5.0–16.2)	6.3 (5.0–11.9)	0.137
Pre-hospital fever days	4.0 (2.0–5.0)	4.0 (2.0–7.0)	3.5 (2.0–5.0)	3.0 (2.0–5.0)	0.221
Corticosteroid use before admission, *n* (%)	52 (14.2)	15 (15.5)	27 (17.8)	10 (8.6)	—

ALT, alanine aminotransferase; CRP, C-reactive protein; EBV, Epstein–Barr virus; IQR, interquartile range; SD, standard deviation; WBC, white blood cell count. Values are presented as median (IQR), mean ± SD, or *n* (%), as appropriate. Between-group comparisons were performed using one-way ANOVA for normally distributed variables, the Kruskal–Wallis test for non-normally distributed variables, and the chi-squared test for categorical variables.

### Association between EBV DNA load and serum ALT

**Figure 2:**
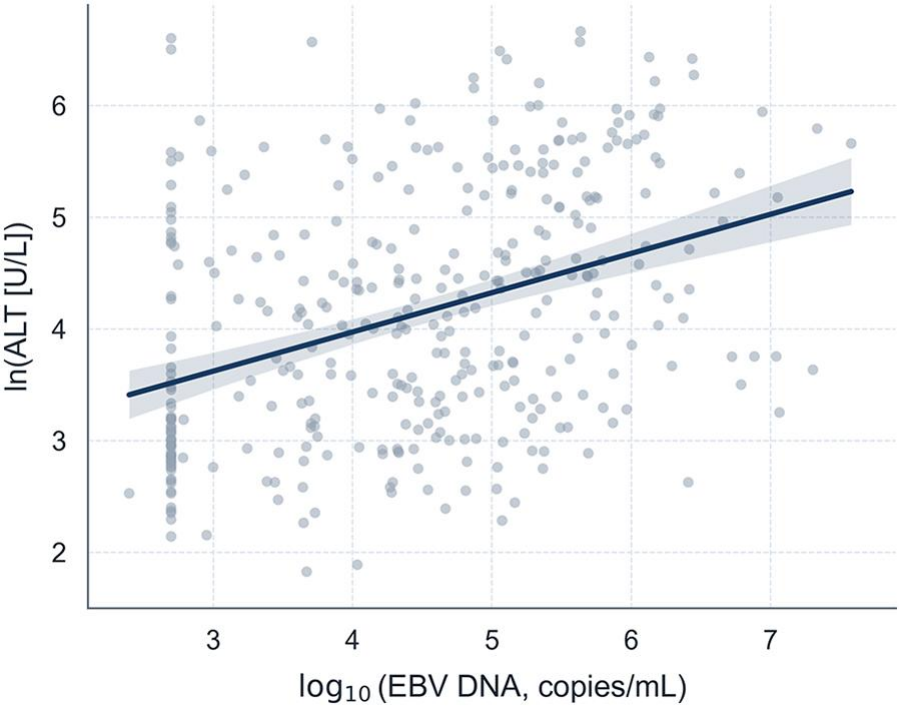
Association between EBV DNA load and serum ln(ALT). Each point represents one patient (*n* = 365). The regression line and shaded band represent the unadjusted linear fit and 95% confidence interval from simple linear regression. Spearman *ρ* = 0.403, *P* < 0.001. ALT, alanine aminotransferase; CI, confidence interval; EBV, Epstein–Barr virus.

Figure 2 shows a positive bivariate association between log10(EBV DNA) and ln(ALT). Higher EBV DNA load was moderately correlated with higher ln(ALT) (Spearman *ρ* = 0.403, *P* < 0.001, *n* = 365). Although ALT values varied across patients at similar EBV DNA levels, the overall positive trend supported further evaluation in sequential multivariable linear regression models (Table 2).

**Table 2:** Multivariable linear regression for the association between EBV DNA load and ln(ALT).

Variable	Model 1 Crude *β* (95% CI)	*P* value	Model 2 Adj. *β* (95% CI)	*P* value	Model 3 Adj. *β* (95% CI)	*P* value
log10(EBV DNA)	0.35 (0.26–0.44)	<0.001	0.32 (0.23–0.40)	<0.001	0.32 (0.24–0.41)	<0.001
Age <3 years						ref
Age 3–6 years	—	—	0.35 (0.09–0.60)	0.008	0.36 (0.10–0.61)	0.007
Age ≥6 years	—	—	0.69 (0.42–0.96)	<0.001	0.70 (0.43–0.97)	<0.001
Female						ref
Male	—	—	−0.51 (−0.73 to −0.29)	<0.001	−0.51 (−0.73 to −0.29)	<0.001
Pre-hospital fever days	—	—	—	—	0.01 (−0.02 to 0.04)	0.471

Model 1 was unadjusted; Model 2 was adjusted for sex and age group; Model 3 was additionally adjusted for pre-hospital fever days and was treated as the primary model. *β* represents the change in ln(ALT) per one-unit increase in log10(EBV DNA), corresponding to a 10-fold increase in EBV DNA load. Reference categories were age <3 years and female sex. All *P* values were two-sided; *n* = 365.

In sequential linear regression models, log10(EBV DNA) was consistently associated with higher ln(ALT) (Table 2). The unadjusted coefficient was *β* = 0.35 (95% CI: 0.26–0.44; *P* < 0.001), and the estimate remained stable after adjustment for sex, age group, and pre-hospital fever days (fully adjusted *β* = 0.32, 95% CI: 0.24–0.41; *P* < 0.001). This fully adjusted estimate corresponds to an approximately 38% higher serum ALT level for each 10-fold increase in EBV DNA load.

Age group and sex were also associated with ln(ALT). Compared with children aged <3 years, those aged 3–6 years (adjusted *β* = 0.36, 95% CI: 0.10–0.61; *P* = 0.007) and ≥6 years (adjusted *β* = 0.70, 95% CI: 0.43–0.97; *P* < 0.001) had higher ln(ALT) in the fully adjusted model. compared with female, male was associated with lower ln(ALT) (adjusted *β* = −0.51, 95% CI: −0.73 to −0.29; *P* < 0.001). Pre-hospital fever days were not significantly associated with ln(ALT).

### Factors associated with ALT elevation: logistic regression

At admission, 212 of 365 patients (58.1%) had ALT elevation (>40 U/L). In the fully adjusted logistic regression model (Model B; Table 3), higher EBV DNA load was associated with greater odds of ALT elevation, with an adjusted OR of 1.91 for each 10-fold increase in EBV DNA load (95% CI: 1.53–2.37; *P* < 0.001). Compared with children aged <3 years, those aged 3–6 years (adjusted OR 2.08, 95% CI: 1.15–3.74; *P* = 0.015) and ≥6 years (adjusted OR 5.89, 95% CI: 3.04–11.42; *P* < 0.001) had higher odds of ALT elevation. Male was associated with lower odds of ALT elevation (adjusted OR 0.29, 95% CI: 0.17–0.51; *P* < 0.001). The estimates were similar in the model without pre-hospital fever days (Model A), suggesting that adjustment for fever duration had little influence on the observed associations. Although children aged 3–6 years had the highest median EBV DNA load, the highest adjusted odds of ALT elevation were observed in children aged ≥6 years. The fully adjusted model showed acceptable discrimination, with a C-statistic of 0.778 (95% CI: 0.731–0.824).

**Table 3:** Multivariable logistic regression for elevated ALT (>40 U/L).

Variable	Crude OR (95% CI)	*P* value	Model A Adj. OR (95% CI)	*P* value	Model B Adj. OR (95% CI)	*P* value
log10 (EBV DNA)	1.84 (1.51–2.24)	<0.001	1.86 (1.51–2.30)	<0.001	1.91 (1.53–2.37)	<0.001
Age <3 years						ref
Age 3–6 years	2.08 (1.24–3.49)	0.006	2.03 (1.13–3.64)	0.018	2.08 (1.15–3.74)	0.015
Age ≥6 years	4.66 (2.59–8.37)	<0.001	5.72 (2.96–11.06)	<0.001	5.89 (3.04–11.42)	<0.001
Female						ref
Male	0.38 (0.23–0.62)	<0.001	0.29 (0.17–0.51)	<0.001	0.29 (0.17–0.51)	<0.001
Pre-hospital fever days	0.97 (0.91–1.04)	0.410	—	—	1.04 (0.96–1.12)	0.328

Model A was a parsimonious model adjusted for log10(EBV DNA), sex, and age group; Model B additionally included pre-hospital fever days and was treated as the primary logistic model. Reference categories were age <3 years and female sex. All *P* values were two-sided; *n* = 365, with 212 events (58.1%).

### Subgroup analysis by age group

**Figure 3:**
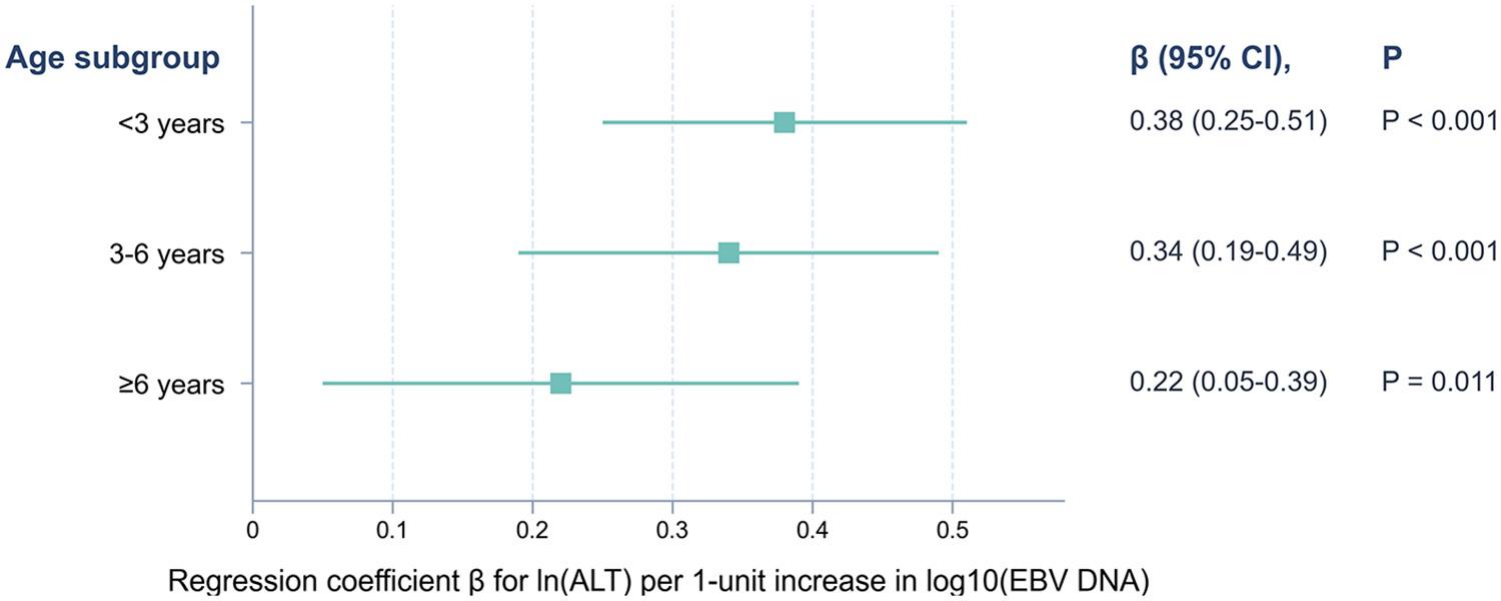
Association between EBV DNA load and ln(ALT) across prespecified age subgroups. Points indicate adjusted regression coefficients and horizontal bars indicate 95% confidence intervals. Models were adjusted for sex and pre-hospital fever days within each age group. The adjusted *P* value for the age group × log10(EBV DNA) interaction was 0.159.

The positive association between log10(EBV DNA) and ln(ALT) was observed across all age groups (Table 4; Figure 3). In models adjusted for sex and pre-hospital fever days within each age group, each 1-unit increase in log10(EBV DNA) was associated with higher ln(ALT) in children aged <3 years (*β* = 0.38; 95% CI: 0.25–0.51; *P* < 0.001), 3–6 years (*β* = 0.34; 95% CI: 0.19–0.49; *P* < 0.001), and ≥6 years (*β* = 0.22; 95% CI: 0.05–0.39; *P* = 0.011). The age group × log10(EBV DNA) interaction was not statistically significant in either the unadjusted model (*P* = 0.321) or the adjusted model (*P* = 0.159), providing no evidence that the association differed significantly across age groups.

**Table 4:** Subgroup analysis of the association between EBV DNA load and ln(ALT) by age group.

Age subgroup	n	Crude *β* (95% CI)	*P* value	Adjusted *β* (95% CI)	*P* value	*P* for Interaction (Crude|Adjusted)
Age <3 years	97	0.38 (0.25–0.51)	<0.001	0.38 (0.25–0.51)	<0.001	0.321|0.159
Age 3–6 years	152	0.36 (0.22–0.51)	<0.001	0.34 (0.19–0.49)	<0.001	—
Age ≥6 years	116	0.23 (0.06–0.40)	0.010	0.22 (0.05–0.39)	0.011	—

Adjusted models included sex and pre-hospital fever days as covariates, with age group omitted within strata. *β* represents the regression coefficient for each one-unit increase in log10(EBV DNA). *P* for interaction was assessed using the log10(EBV DNA) × age-group interaction term and is reported once for the subgroup comparison.

### Sensitivity analyses

Redefining ALT elevation using age- and sex-specific pediatric upper limits of normal did not materially change the primary findings (Supplementary Table 1). In the two alternative-threshold models, log10(EBV DNA) remained independently associated with ALT elevation (adjusted OR 2.07, 95% CI: 1.64–2.61; and adjusted OR 2.05, 95% CI: 1.65–2.55; both *P* < 0.001). The associations of age group and sex were consistent with the primary analysis, whereas pre-hospital fever days remained non-significant.

Additional adjustment for pre-admission corticosteroid use also yielded estimates consistent with the primary models (Table 5). The association between log10(EBV DNA) and ln(ALT) was unchanged (adjusted *β* = 0.32, 95% CI: 0.24–0.41; *P* < 0.001), and the logistic sensitivity model produced an estimate nearly identical to the primary Model B result for ALT elevation (adjusted OR 1.91, 95% CI: 1.54–2.38; *P* < 0.001). Pre-admission corticosteroid use was not significantly associated with either ln(ALT) or ALT elevation. In age-stratified analyses, the EBV DNA–ALT associations remained statistically significant in all three age groups, and corticosteroid adjustment did not materially change the interaction result (crude and adjusted interaction *P* values: 0.337 and 0.161, respectively; Table 5).

**Table 5:** Sensitivity analyses with additional adjustment for pre-admission corticosteroid use.

Variable/analysis	Primary model estimate (95% CI)	*P* value	Sensitivity model (+corticosteroid) estimate (95% CI)	*P* value
A. Linear regression [outcome: ln(ALT)]				
log10(EBV DNA)	0.32 (0.24–0.41)	<0.001	0.32 (0.24–0.41)	<0.001
Male	−0.51 (−0.73 to −0.29)	<0.001	−0.50 (−0.73 to −0.28)	<0.001
Age 3–6 years	0.36 (0.10–0.61)	0.007	0.36 (0.10–0.62)	0.006
Age ≥6 years	0.70 (0.43–0.97)	<0.001	0.69 (0.42–0.96)	<0.001
Pre-hospital fever days	0.01 (−0.02–0.04)	0.471	0.01 (−0.02–0.05)	0.391
Corticosteroid use before admission (yes vs. no)	—	—	−0.13 (−0.43–0.17)	0.395
B. Logistic regression (outcome: elevated ALT >40 U/L)				
log10(EBV DNA)	1.91 (1.53–2.37)	<0.001	1.91 (1.54–2.38)	<0.001
Male	0.29 (0.17–0.51)	<0.001	0.30 (0.17–0.52)	<0.001
Age 3–6 years	2.08 (1.15–3.74)	0.015	2.10 (1.17–3.78)	0.013
Age ≥6 years	5.89 (3.04–11.42)	<0.001	5.80 (2.99–11.26)	<0.001
Pre-hospital fever days	1.04 (0.96–1.12)	0.328	1.04 (0.97–1.13)	0.274
Corticosteroid use before admission (yes vs. no)	—	—	0.78 (0.40–1.52)	0.462
C. Subgroup linear analysis [outcome: ln(ALT)]				
Age <3 years	0.38 (0.25–0.51)	<0.001	0.38 (0.25–0.51)	<0.001
Age 3–6 years	0.34 (0.19–0.49)	<0.001	0.34 (0.19–0.49)	<0.001
Age ≥6 years	0.22 (0.05–0.39)	0.011	0.24 (0.07–0.41)	0.007
*P* for interaction (crude|adjusted)	—	0.321|0.159	—	0.337|0.161

The primary linear and logistic model included log10(EBV DNA), sex, age group, and pre-hospital fever days; Sensitivity models additionally included pre-admission corticosteroid use. Female and age <3 years were used as the reference categories in Models A and B. In subgroup analyses, adjusted *β* (95% CI) and *P* values for log10(EBV DNA) are reported within each age stratum. In the corticosteroid sensitivity subgroup analysis, the crude and adjusted interaction *P* values were 0.337 and 0.161, respectively. All estimates shown are adjusted unless otherwise specified.

In continuous-age sensitivity analyses, replacing categorical age groups with continuous age did not materially change the association between log10(EBV DNA) and ln(ALT) (adjusted *β* = 0.32 in both Model A and Model B; Supplementary Table 2). Continuous age was independently associated with ln(ALT), whereas pre-hospital fever days remained non-significant. Consistent with the continuous dose–response observed in the primary linear models (Table 2), a *post hoc* ordinal analysis categorizing ALT by multiples of the ULN (≤1×, 1–5×, and >5×) confirmed that each 10-fold increase in EBV DNA load was associated with higher odds of more severe ALT elevation (adjusted OR: 1.82, 95% CI: 1.52–2.19, *P* < 0.001; Supplementary Table 3).

In an exploratory clinical-course analysis, a high EBV DNA load (>10⁶ copies/mL) was associated with a longer hospital stay and more in-hospital fever days (Supplementary Table 4).

To address possible temporal variation in PCR assays, we performed an admission-period sensitivity analysis. EBV DNA load did not differ significantly between 2017 and 2021 and 2022–2025 (median log10 EBV DNA 4.46 vs. 4.54; *P* = 0.39), and adjustment for admission period did not change the EBV DNA–ln(ALT) association (adjusted *β* = 0.32, 95% CI: 0.23–0.40). Period-stratified estimates were concordant, with no significant period × log10(EBV DNA) interaction (*P* = 0.84; Supplementary Table 5).

## Discussion

In this single-center retrospective cross-sectional study of 365 hospitalized children with EBV-associated IM, higher admission EBV DNA load was associated with higher ALT in all regression models. Each 10-fold increase in EBV DNA load was associated with an estimated 38% higher serum ALT (95% CI: 27%–51%), and the estimate remained stable after additional adjustment for pre-admission corticosteroid use. Older age and female sex were also independently associated with ALT elevation, whereas pre-hospital fever duration and pre-admission corticosteroid use did not show independent associations with ALT outcomes in the adjusted or sensitivity models. Children aged ≥6 years had the highest odds of ALT elevation despite lower median EBV DNA load than children aged 3–6 years, this age-related pattern is broadly consistent with previous pediatric cohorts reporting more frequent or more pronounced liver enzyme abnormalities in older children with primary EBV infection (5, 8, 26).

The observed dose-related association between higher EBV DNA load and higher ALT is biologically plausible but should not be interpreted as evidence of a direct causal mechanism. Higher EBV DNA load may instead reflect greater host-virus immune activation associated with ALT elevation, rather than a direct effect of viral replication on hepatocellular injury. Studies of EBV immune dynamics have described CD8+ T-cell expansion, natural killer cell involvement, and broader immune activation during acute IM (27–29). More directly, recent pediatric studies have linked higher EBV DNA load or immune signatures with hepatic dysfunction or liver injury in pediatric IM (15, 22, 30, 31). These findings provide biological context for the present association, but they do not establish the mechanism in our cohort. Because EBV DNA and ALT were measured at admission, temporal direction and causal mediation cannot be inferred.

The positive association between EBV DNA load and ln(ALT) was observed in all three age groups, supporting the robustness of the main finding across age groups. Interaction testing did not show statistically significant evidence that this association differed by age group. However, because the estimate was numerically smaller in children aged ≥6 years, moderate age-related heterogeneity cannot be excluded. Together with the pediatric evidence discussed above linking EBV DNA load and immune-related markers with hepatic dysfunction or liver injury, these findings suggest that EBV DNA load may provide useful context for interpreting ALT elevation in hospitalized children with IM, but should be considered alongside other clinical and laboratory information (15, 22, 30, 31). The association also persisted when ALT elevation was redefined using age- and sex-specific pediatric reference thresholds, suggesting that the primary binary findings were not driven by the fixed 40 U/L cutoff alone.

Age ≥6 years showed the strongest association with ALT elevation in the primary logistic model, despite lower median EBV DNA load than in children aged 3–6 years. This age–viral-load discordance suggests that measured EBV DNA load alone may not fully capture age-related differences in clinical phenotype, host response, or other unmeasured factors relevant to ALT elevation (22, 25, 26, 31). This context may help interpret the observed age pattern, but the interpretation remains hypothesis-generating because immune phenotyping was not available in the present study. If confirmed, this pattern suggests that a given EBV DNA load may need to be interpreted in the context of age, because older children may have a higher propensity for ALT elevation even when measured viral load is not the highest. This interpretation remains hypothesis-generating and should not be taken as definitive evidence that age modifies the EBV DNA–ALT association, but it supports future studies of age-stratified monitoring approaches.

Restricted cubic spline analysis suggested an approximately linear increase in ln(ALT) with age across the observed range, without evidence of nonlinearity. The prespecified age groups are useful for clinical description and interpretation, but the cutpoints should not be interpreted as discrete biological thresholds. When age was modeled as a continuous variable, the EBV DNA–ALT association was virtually unchanged, further supporting the stability of the primary association between EBV DNA load and ALT.

Male sex was associated with lower odds of ALT elevation in the primary adjusted model, corresponding to higher odds among female children in this cohort. This finding may reflect unmeasured sex-related differences in host response, but our study did not measure sex-specific immune mediators or test the interaction between EBV DNA and sex. Therefore, the sex association should be interpreted as hypothesis-generating rather than as evidence that sex modifies the EBV DNA-ALT relationship. Broader antiviral immunology literature provides possible biological context, including sex differences in innate and adaptive immune responses and X-linked immune regulatory pathways such as Toll-like receptor 7 signaling (20, 21, 32), but these mechanisms were not evaluated in our cohort. At this stage, we consider the sex association insufficiently characterized to support sex-specific monitoring thresholds or other clinical recommendations; however, given that female children had substantially higher odds of ALT elevation, this finding warrants validation in prospective cohorts and may inform the design of future monitoring studies.

Neither pre-hospital fever duration nor pre-admission corticosteroid use showed an independent association with ALT outcomes after adjustment for EBV DNA load, age group, and sex. This finding suggests that these variables provided limited explanatory information in the available retrospective data. However, the absence of an observed association with corticosteroid use should not be interpreted as evidence of no treatment effect, because corticosteroid exposure was non-randomized and incompletely characterized, and may have been influenced by indication, illness severity, and local practice patterns.

In an exploratory analysis, a high admission EBV DNA load (>10^6^ copies/mL) was associated with an approximately 26% longer hospital stay and 57% more in-hospital fever days than a low load (Supplementary Table 4). This finding is consistent with a prior pediatric report linking viral burden to duration of hospitalization (16); nonetheless, given the small high-load subgroup and the observational design, this observation is hypothesis-generating rather than evidence of a clinically actionable predictor.

Although ALT elevation was defined biochemically rather than as clinically diagnosed hepatitis, the supplementary analyses help place this outcome in clinical context. In a proportional-odds ordinal regression, each 10-fold increase in EBV DNA load was associated with 1.82-fold higher odds of being in a more severe ALT category (≤1×, 1–5×, or >5× ULN; Supplementary Table 3). Descriptively, median ALT increased across EBV DNA groups from 29 U/L in children with <10^4^ copies/mL to 73 U/L in those with 10^4^–10^6^ copies/mL and 160 U/L in those with >10^6^ copies/mL; the high EBV DNA load group also had a modestly longer hospital stay and more fever days (Supplementary Table 4). Because these clinical-course indicators were analyzed by EBV DNA load group rather than directly by ALT status, the relationship between ALT elevation and hospital course should be interpreted indirectly through their shared association with EBV DNA load. Because ALT is already available at the same admission timepoint as EBV DNA, the value of these associations lies not in predicting an unmeasured outcome but in contextualizing the degree of biochemical liver involvement within the admission viral-load profile and in generating testable hypotheses about hepatic trajectory beyond the index hospitalization. ALT elevation in this cohort should therefore be viewed as a marker of biochemical liver involvement that tracks with viral burden and may inform monitoring intensity, rather than as a direct determinant of clinical course or evidence of severe liver disease.

Several limitations should be acknowledged. First, this was a single-center retrospective cross-sectional study restricted to hospitalized children with quantitative blood EBV DNA results, which limits generalizability and likely reflects a clinically selected subgroup of pediatric IM. The 58.1% ALT elevation rate observed in this cohort should therefore be interpreted in the context of hospitalized patients rather than extrapolated to all children with IM. Second, EBV DNA and ALT were measured concurrently at admission, so the temporal sequence of viral burden and ALT elevation could not be established, and the direction of the association cannot be inferred. Third, although a uniform ALT cutoff of 40 U/L was applied in the primary binary analysis for clinical consistency, pediatric ALT reference ranges vary by age, sex, and laboratory standards; sensitivity analyses using age- and sex-specific pediatric thresholds yielded consistent results, although residual misclassification cannot be excluded. Fourth, ALT was the only hepatic marker analyzed; other indicators of liver involvement, including bilirubin, *γ*-glutamyltransferase, alkaline phosphatase, and hepatic synthetic-function indices, were not uniformly available across the retrospective study period and therefore could not be incorporated. The hepatic outcome in this study should accordingly be understood as reflecting ALT-based biochemical injury rather than a comprehensive assessment of liver disease. Fifth, residual confounding from unmeasured factors, including illness severity, coexisting infections, prior medications, host immunologic profile, and EBV genotype, cannot be excluded. Sixth, detailed assay-platform information was not consistently recoverable from retrospective records across the full study period, limiting formal harmonization across assay versions. Finally, the lack of granular data on corticosteroid dose and timing precluded causal interpretation of steroid-related effects. With respect to assay variability, however, the distribution of EBV DNA load and the EBV DNA–ALT association were both stable across admission periods, and adjustment for period did not change the estimate (Supplementary Table 5), arguing against material confounding from temporal changes in assay methodology, even though this analysis assesses temporal stability rather than direct cross-platform calibration. In view of these constraints, and because the exposure and outcome were measured concurrently, the present findings should be regarded as hypothesis-generating, primarily descriptive associations rather than evidence of causal, temporal, or clinically actionable relationships.

## Conclusion

Among hospitalized children with EBV-associated IM, higher EBV DNA load, older age, and female sex were independently associated with ALT elevation, findings that may help contextualize ALT elevation at initial evaluation, particularly in older children, female children, and those with a high EBV DNA load. These subgroups generate specific hypotheses for prospective testing: whether a given EBV DNA load carries different implications for ALT elevation across age groups, and whether the EBV DNA–ALT gradient translates into prognostic information beyond the index hospitalization.

## Data Availability

Due to patient privacy and ethical restrictions, the datasets generated and/or analyzed during the current study are not publicly available but are available from the corresponding author on reasonable request and with permission of the institutional ethics committee. Requests to access these datasets should be directed to Songlin Gan, songlingan92@163.com.
