# Supplementary figures and images for: Alanine aminotransferase elevation in hospitalized children with infectious mononucleosis: independent associations with Epstein–Barr virus DNA load, age, and sex
Source: Front Pediatr. 2026 Jul 7;14:1901064. doi: 10.3389/fped.2026.1901064 (PMC13385180; doi:10.3389/fped.2026.1901064)

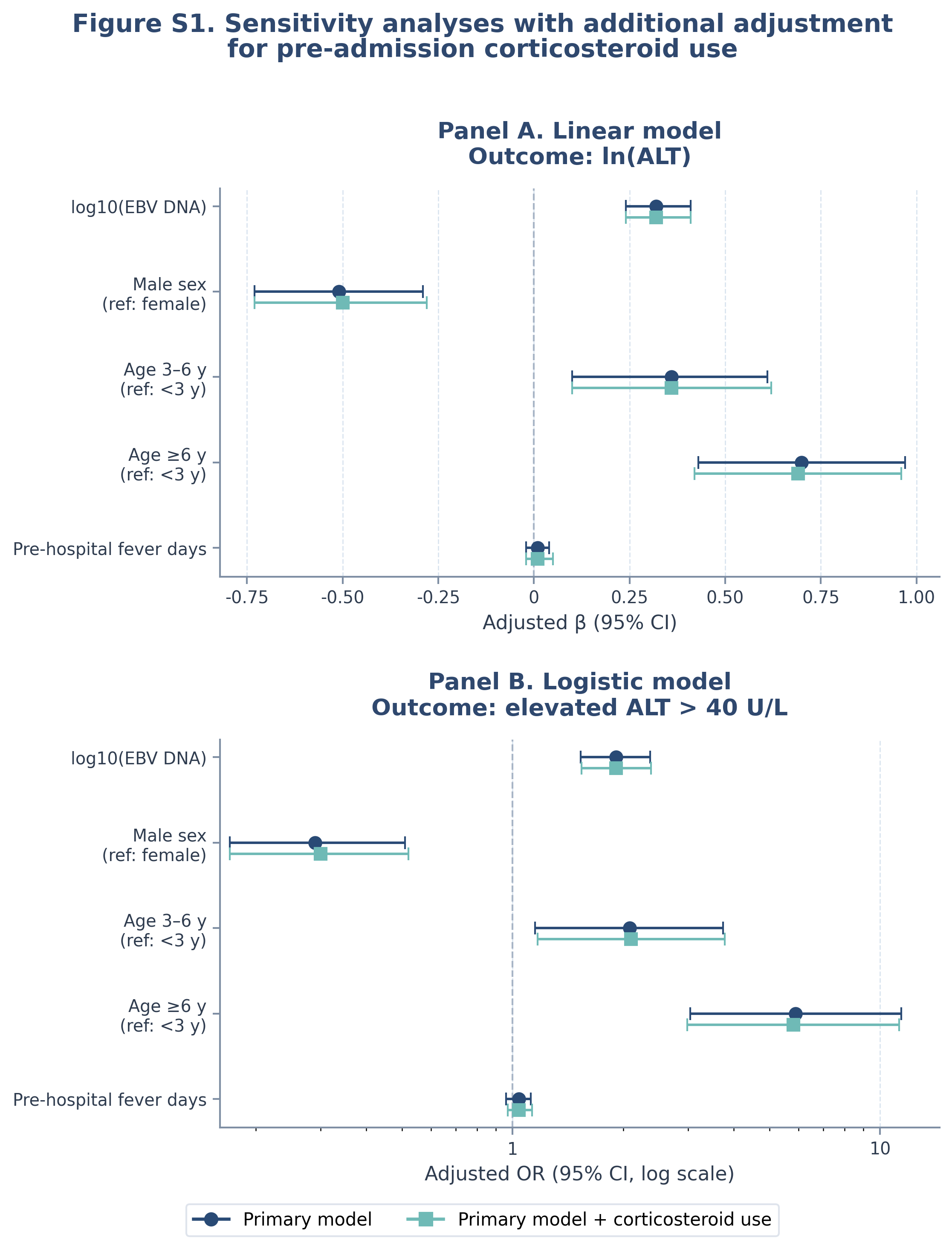

Supplement: Supplementary file 1 [file Image1.png]

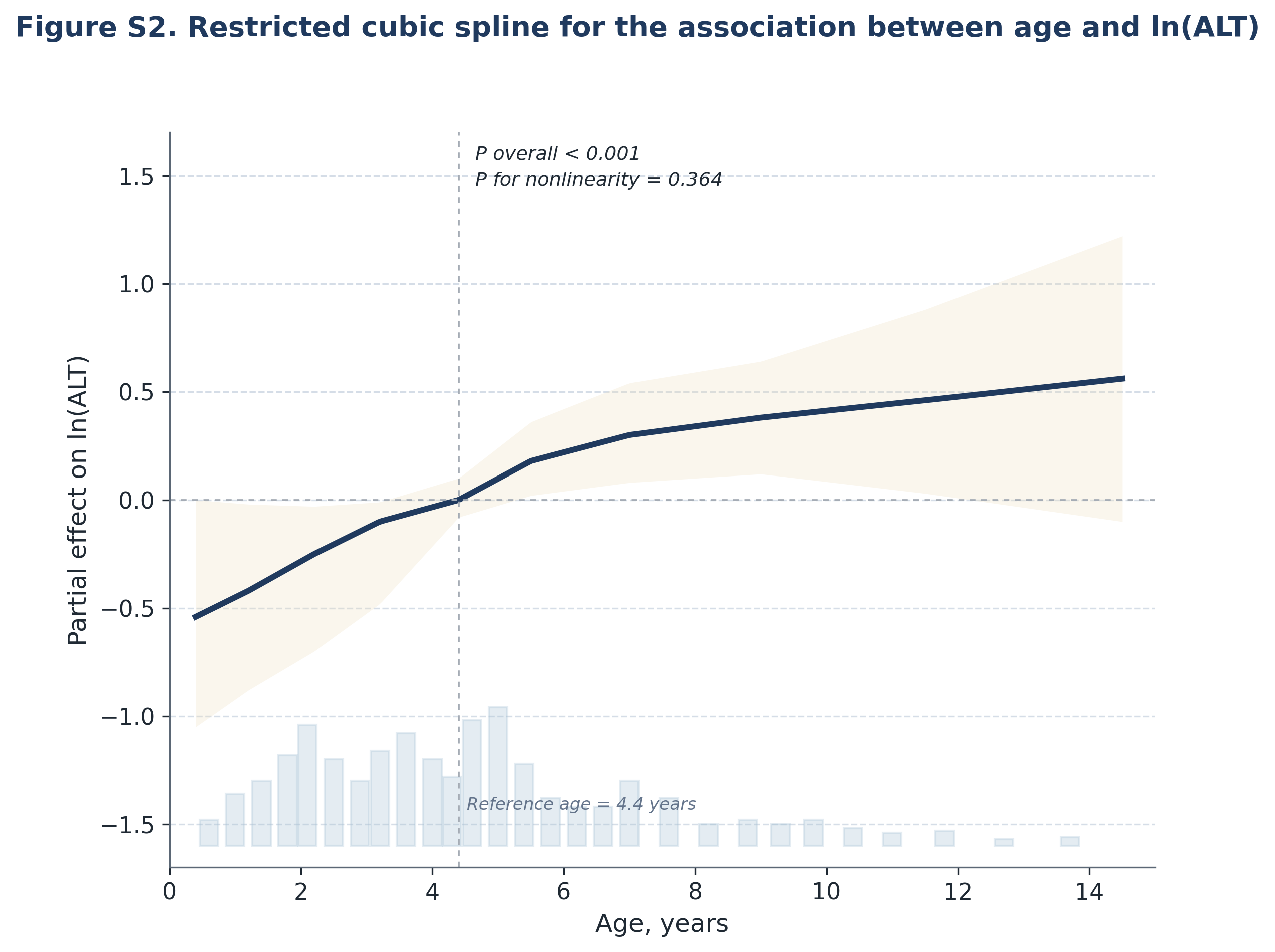

Supplement: Supplementary file 2 [file Image2.png]
